# Phase II Trial of Pure Hypofractionated Radiotherapy in the Treatment of Localized Carcinoma of the Prostate

**DOI:** 10.7759/cureus.3435

**Published:** 2018-10-09

**Authors:** Darin Gopaul, Dilip Panjwani, Robert F Stephens, Michael Lock

**Affiliations:** 1 Radiation Oncology, Grand River Regional Cancer Centre, Kitchener, CAN; 2 Radiation Oncology, Prince Edward Island Cancer Treatment Centre, Charlottetown, CAN; 3 Radiation Oncology, University of Toronto, Toronto, CAN; 4 Radiation Oncology, Schulich School of Medicine & Dentistry - Western University, London, CAN

**Keywords:** conformal radiotherapy, hypofractionation, prostate cancer, phase ii, clinical trial

## Abstract

Purpose

To evaluate acute and late genitourinary (GU) and gastrointestinal (GI) toxicity and the biochemical control of pure hypofractionated radiotherapy (without acceleration) for the treatment of prostate cancer.

Methods and materials

This phase II prospective trial evaluated low-risk and intermediate-risk prostate cancer patients who received hypofractionated radiotherapy. Fifty-three patients with low-risk prostate cancer received 50 Gy in 15 fractions, 156 patients with intermediate-risk prostate cancer received 60 Gy in 20 fractions over eight weeks. Acute toxicity and late toxicity were graded per the Radiation Therapy Oncology Group (RTOG) toxicity scales and the Phoenix Definition (nadir plus two) defined biochemical failure.

Results

Median follow-up was 6.5 years. Acute phase grade 2/3 toxicity was 6%/0 and 8%/2% for GI and GU symptoms, respectively, and one grade 4 acute GU toxicity (0.5%). Late grade 2/3 GI and GU toxicity were 7%/0 and 8%/0.5%, respectively. There were no late grade 4 toxicities. The five-year freedom-from-biochemical-failure (FFBF) rates were 85% for low-risk patients and 80% for intermediate-risk patients.

Conclusions

Pure hypofractionation seems to be associated with low toxicity rates and biochemical control rates that are similar or better than those observed with accelerated hypofractionated or conventionally fractionated therapy.

## Introduction

Hypofractionated radiation therapy involves delivering fewer fractions of radiation at a higher dose per fraction. This is appealing from the point of view of patients, for resource utilization, and for third-party payers. In contrast to most other tumors, there is evidence to suggest that the α/β for prostate cancer is lower than or similar to the surrounding late-responding normal tissue [1-7]. If this is the case, hypofractionation is expected to be at least as efficacious as conventional fractionation in terms of tumor control [8-17]. There are two methods of using hypofractionation. The most common method of hypofractionation in prostate cancer is to shorten the overall treatment time (as compared to standard fractionation) along with the hypofractionation. This is called ‘accelerated hypofractionation.’ The second method is to hypofractionate but maintain the same overall treatment time as the standard fractionation. This is called ‘pure hypofractionation’ and treatments are often given two to three times per week instead of daily. Given that the potential doubling time of rectal and bladder mucosa is a few days, accelerated hypofractionation was first hypothesized and then shown in large randomized trials to have an adverse impact on acute gastrointestinal (GI) and genitourinary (GU) toxicity [8,10,16,18-22]. As the potential doubling time of prostate cancer clonogens is several weeks, the use of accelerated hypofractionation does not correspondingly increase the cell kill for the cancer. We, therefore, hypothesized that by keeping the overall treatment time the same as standard fractionation (i.e. by not introducing an element of acceleration along with the hypofractionation, but instead maintaining 'pure' hypofractionation), we would reduce the acute and late toxicity seen in hypofractionation protocols, without impairing the tumor control.

## Materials and methods

Men diagnosed with low or intermediate risk localized prostate cancer were eligible for this Phase II trial [20]. Exclusion criteria included: histologic disease ≥ Gleason 8, prostate-specific antigen levels (PSA) > 20 ng/mL, any previous cytotoxic therapy or androgen deprivation therapy (ADT), estimated life expectancy < 10 years, prior or active malignancy other than non-melanoma skin cancer, any prior pelvic or abdominal radiotherapy, Eastern Cooperative Oncology Group (ECOG) performance status > 1, inflammatory bowel disease, or a pelvic kidney.

Pre-treatment evaluations included a transrectal ultrasound-guided biopsy, histologic confirmation of disease, and Gleason scoring. Laboratory studies included a serum PSA measurement within four months prior to the start of radiation therapy. All patients were required to have a diagnostic or planning computed axial tomography (CT) scan of the pelvis within 10 weeks of the start of treatment. For those patients with PSA > 15 or Gleason 7, a bone scan was required within four months of treatment.

All patients were CT simulated and treated in the supine position with a full bladder and an empty rectum. The planning target volume (PTV) was defined as the clinical tumor volume (CTV) plus a 1.0 cm margin in all directions except posteriorly, where a 0.7 cm margin was applied. The PTV was covered by the 95% isodose line. Image-guided radiotherapy (IGRT) was used in all cases. Radio-opaque fiducial markers and orthogonal kV-kV matching, or cone beam CT (CBCT) localization, was used. Of the patients, 66% were treated with three-dimensional conformal radiotherapy (3D-CRT), and all patients treated after 2008 (34%) were treated with IMRT.

Low-risk patients received a total dose of 50 Gy in 15 fractions (equivalent to 70 Gy in 35 fractions) with two fractions per week while intermediate risk patients received a total of 60 Gy in 20 fractions (equivalent to 78 Gy in 39 fractions) alternating two to three fractions per week over eight weeks. Adjuvant or neoadjuvant androgen deprivation (ADT) was not used. The protocol was approved by our institutional research ethics board. Written informed consent was obtained from all participants.

Follow-up and outcomes

Patients were assessed weekly during radiation therapy, three months after completion of radiotherapy, every six months for five years, and then annually. The acute period was defined as during treatment and three months after the completion of treatment, and the late period was defined as six months after completing treatment and beyond. Acute and late GU and GI toxicity grading were performed at each follow-up in accordance with the Radiation Therapy Oncology Group (RTOG) toxicity scales. The patients were categorized according to their highest toxicity value throughout the acute follow-up period and the late follow-up period. Biochemical failure (BF) was defined according to the Phoenix definition of a PSA nadir plus two. The presence of progression to metastatic disease was also included in the analysis.

Statistical analysis

Data management and statistical analysis were performed using Microsoft Office Excel 2007 (Microsoft, Redmond, WA, US) and IBM SPSS Statistics Version 21.0 (IBM Corporation, North Castle, NY, US). All time-to-event outcomes were summarized using the Kaplan-Meier method [21]. Time-to-event outcome comparisons were made with the Log Rank (Mantel-Cox) statistic with a critical value of α = 0.05.

## Results

Trial population

Two hundred and nine patients (53 low risk and 156 intermediate) were treated between September 2005 and May 2009. The median follow-up time was 6.5 years. The demographics and clinical characteristics for this patient population are summarized in Table 1.

**Table 1:** Demographics of the population

Age (Years)		
Mean ± SD (Range)	71 ± 6 (52-87)	
Population by Risk		
Low	53	
Intermediate	156	
	Low	Intermediate
Initial PSA (ng/ml)		
Mean ± SD (Range)	6.23 +/- 2.2 (1.1-9.7)	8.79 +/- 3.8 (2.3-20.0)
T-Stage		
T1	42	66
T2	11	90
Gleason Score		
6	53	33
7	N/A	123
Biochemical Failure (BF)		
Mean (Months) ± SD (Range)	50.0 +/- 23.6 (18-90)	51.3 +/- 24.4 (0-108)
Total Number of Cases	9	45
Patients with Mets.		
Mean (Months) ± SD (Range)	30.0 +/- 33.9 (6-54)	44.2 +/- 30.3 (12-102)
Total Number of Cases	2	9
Death		
Mean (Months) ± SD (Range)	50.6 +/- 26.4 (6-54)	54.0 +/- 26.9 (0-96)
Total Number of Cases	14	36
Patients Lost to Follow-Up	2	4
Median Follow-Up in Months (Range)	84 (6-108)	78 (0-120)
BF = PSA of nadir + 2 PSA = Prostate-specific antigen Risk = D'Amico risk category		

Acute toxicity

During the acute period, the incidence of GU toxicity events were as follows: 127 (61%) grade 1, 17 (8%) grade 2, 5 (2%) grade 3, and 1 (0.5%) grade 4. The grade 4 toxicity was an acute event of urinary retention. GI toxicity events included 108 (52%) grade 1, 12 (6%) grade 2, and no grade 3 or 4 toxicities. There were a total 12 (6%) patients that experienced acute GI toxicity of RTOG grade ≥2 and 23 (11%) patients that experienced acute GU toxicity of RTOG grade ≥2. All the grade ≥2 GI events were grade 2, and 74% of the GU events were grade 2. There were four (8%) low-risk patients that experienced GU toxicity ≥ grade 2 and three (6%) low-risk patients that experienced GI toxicity ≥ grade 2. Of the intermediate risk patients, 14 (9%) experienced GU toxicity ≥ grade 2 and 9 (6%) experienced GI toxicity ≥ grade 2.

Late toxicity

The occurrence of RTOG late GU symptoms included 25 grade 1 toxicity events (12%), 17 grade 2 toxicity events (8%), and one grade 3 toxicity event (0.5%). There were no grade 4 late GU toxicity events. There were 66 (32%) grade 1 late GI events and 15 (7%) grade 2 events; there were no grade 3 or grade 4 late GI events. There was a total of 18 late GU toxicity ≥ 2 grade events (9%) and 15 late GI toxicity ≥ grade 2 events (7%). Of the low-risk patients, three patients (6%) experienced late GI toxicity ≥ grade 2 and six experienced late GU toxicity ≥ grade 2 (3%). In the intermediate group, there were 12 late GI toxicity events and 12 late GU toxicity events (8% each).

A summary of acute and late toxicity events greater than or equal to RTOG grade 2 can be found in Table 2.

**Table 2:** Acute and late toxicity by RTOG grade RTOG: Radiation Therapy Oncology Group

		RTOG Grade				
		0	I	II	III	IV
Acute Toxicity	Genitourinary % (no. of patients)	29(60)	61 (127)	8 (17)	2 (5)	0.5 (1)
	Gastrointestinal % (no. of patients)	43 (90)	52 (108)	6 (12)	_	_
Late Toxicity	Genitourinary % (no. of patients)	80 (166)	12 (25)	8 (17)	0.5 (1)	_
	Gastrointestinal % (no. of patients)	61 (128)	32 (66)	7 (15)	_	_

**Figure 1:**
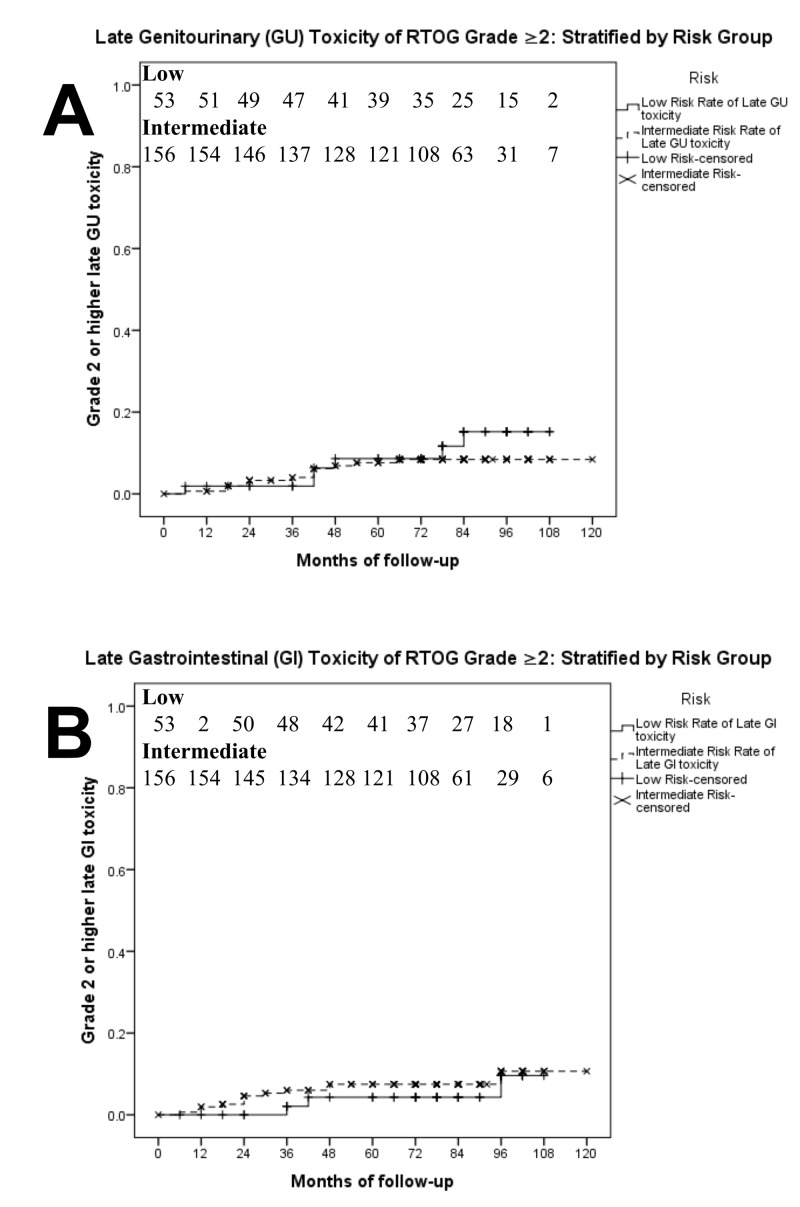
Late grade 2 or 3 gastrointestinal (GI) toxicity (A) and genitourinary (GU) toxicity (B) in men who received hypofractionated radiotherapy (n = 209) stratified by risk group, low (n = 53) and intermediate (n = 156)

The results of an actuarial analysis for the cumulative incidence of late toxicity events ≥ RTOG grade 2 can be found in Figure 1. The five-year cumulative incidence of GI toxicity for the overall study population was 7%. For low-risk patients, the five-year incidence of GI toxicity was 4%, and for intermediate-risk patients, it was 8%. The difference between the low and intermediate risk groups was not statistically significant (p > 0.05). The overall five-year cumulative incidence of GU toxicity was 8%; 9% for the low-risk group and 8% for the intermediate risk group. The difference between the five-year rates observed for the low- and intermediate-risk groups was not significant (p > 0.05).

Biochemical failure and metastatic disease

**Figure 2:**
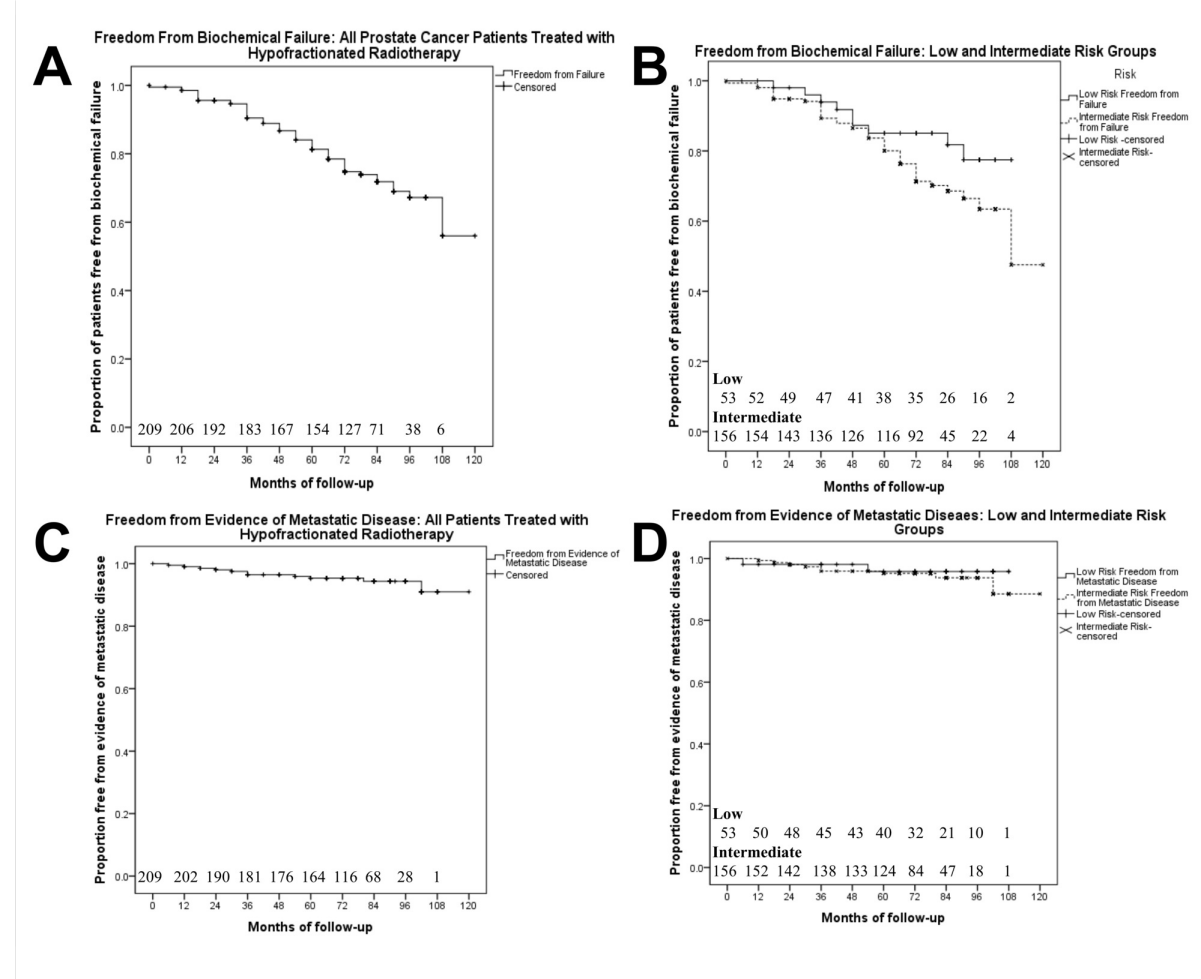
Freedom from biochemical failure using the Phoenix Definition (A, B) and freedom from evidence of metastatic disease (C, D) in men treated with hypofractionated radiotherapy (n = 209) in the total trial population (A, C) and stratified into low (n = 53) and Intermediate (n = 156) risk groups (B, D)

At the time of analysis, 54 patients (26%) had experienced BF; nine had a low-risk disease and 45 had an intermediate-risk disease. Further patient characteristics and clinical information regarding disease progression can be found in Table 1. The freedom-from- biochemical failure (FFBF) curves can be found in Figures 2A-2B. The five-year FFBF rate for the study population was 81%. The five-year FFBF rates for low-risk patients was 85%, and for the intermediate-risk group, the five-year FFBF was 80%. The difference between the risk groups was not statistically significant (p > 0.05), but there appears to be a divergence between the curves beginning at five years. The actuarial results for freedom-from-metastatic-disease are found in Figures 2C-2D. All patients with metastatic disease (11) experienced BF before progression.

## Discussion

In recent years, hypofractionation has been gaining acceptance in the literature. Jack Fowler popularized the theory that prostate cancer has a low α/β. An α/β of 1.5 would suggest that a regimen of 60 Gy in 20 fractions should have biochemical control and toxicity profiles similar to a standard schedule of 78 Gy in 39 fractions, so we chose to use this regimen with our intermediate patients [22-23]. At the time the study protocol was written, there was limited evidence for dose-escalated radiotherapy in low-risk patients and 70 Gy in 35 fractions was our institutional standard. The equivalent dose of 50 Gy in 15 fractions was chosen for low-risk patients.

So far, four, large randomized trials have presented results supporting the hypothesis that hypofractionated radiotherapy is not inferior to conventional fractionation in terms of biochemical control (Table 3). However, two of these trials reported a higher incidence of late toxicity with hypofractionated radiotherapy [8,24]. Recently, however, the prostate fractionated irradiation (PROFIT) trial found that hypofractionated radiotherapy provided better rates of late GI toxicity compared to the conventional fractionation schedule [14]. These trials used accelerated hypofractionation regimens, decreasing the treatment time from seven or eight weeks to four or five weeks. In this phase II study, we find support for the hypothesis that pure hypofractionated radiotherapy (delivered over seven to eight weeks) allows for low rates of acute and late toxicity events while maintaining excellent biochemical control. The potential doubling time of prostate cancer cells is much longer than that of rectal and bladder mucosa, and hence we investigated the therapeutic gain in terms of reducing the acute and consequential late effects of radiotherapy [25]. In particular, consequential late effects may be the predominant component of late toxicity in the rectum [19].

**Table 3:** Summary of outcomes from hypofractionation trials For randomized studies, the order of reported values is as follows: values from the hypofractionated regimen are reported first, values from the conventional fractionation reported second. FU = Median follow-up time for all patients, in years. Treatment technique: IMRT = intensity modulated radiotherapy; 3D-CRT = 3- dimensional conformal radiotherapy. FFBF = Freedom from biochemical failure, according to the Phoenix definition of biochemical failure, which is a PSA greater than nadir + 2 Toxicity Scoring: RTOG = the Radiation Therapy Oncology Group Late Toxicity Scale; NCIC = National Cancer Institute common terminology criteria for adverse effects*; *GI = gastrointestinal; GU = genitourinary; N.R. = Not reported; PROFIT: Prostate fractionated irradiation; HYPRO: Hypofractionated irradiation for prostate cancer; CHHiP: Conventional or hypofractionated high dose intensity modulated radiotherapy for prostate cancer; MDACC: MD Anderson Cancer Center ** = From Kuban et al. 2016. (Abstract) Gopaul et al. is the current study.

Study	n	FU	Treatment Technique	Treatment Dose Regimen	FFBF (years)	Toxicity Scoring	Late GI Toxicity ≥ Grade 2 (years)	Late GU Toxicity ≥ Grade 2 (years)
PROFIT	1206	6	IMRT	60 Gy/ 20 fx vs. 78 Gy/ 39 fx	85% vs. 85% (5)	RTOG	8.9% vs. 13.9% (5)	22% vs. 22% (5)
HYPRO	820	5	IMRT	64.6 Gy/19 fx vs. 78 Gy/39 fx	N.R.	RTOG	21.9% vs. 17.7% (3)	19.0% vs. 12.9% (3)
CHHiP	3216	5.2	IMRT	57 Gy/ 19 fx vs. 60 Gy/ 20 fx vs. 74 Gy/ 37 fx	85.9% vs. 90.6% vs. 88.3% (5)	RTOG	11.3% vs. 11.9% vs. 13.7% (5)	6.6% vs. 11.7% vs. 9.1% (5)
MDACC	203	6	IMRT	72 Gy/ 30 fx vs. 75.6 Gy/ 42 fx	89.3% vs. 84.6% (8) **	RTOG	10% vs. 5.1% (5)	15.8% vs. 16.5% (5)
Lieng et al. [26]	96	10.6 vs. 9	IMRT	60 Gy/ 20 fx vs. 66 Gy/ 22 fx	66% vs. 80% (5)	RTOG	4% vs. 21% (8)	12% vs. 4% (8)
Lock et al. [16]	66	3	IMRT	63.2 Gy/ 20 fx	95% (3)	NCIC	28.1% (3)	20.3% (3)
Gopaul et al.	209	6.5	3DCRT/ IMRT	60 Gy/20 fx (int.) 50 Gy/15 fx (low)	81% (5)	RTOG	6.7% (5)	7.9% (5)

Acute toxicity

With accelerated hypofractionation and conventional fractionation schedules, there are 24 hours between fractions. Our pure hypofractionation schedule allows for 48-72 hours for interfraction repair. Our rate of acute toxicity (Table 2) was low, with 11% reporting grade > 2 GU toxicity (including one grade 4 acute GU toxicity) and 6% reporting grade ≥ 2 GI toxicity (no grade 3 or 4). A recent Canadian trial in London also investigated a 20-fraction regimen (Lock et al. in Table 3). Acute Grade ≥2 toxicity was 43% for GU and 36% for GI symptoms [16]. Importantly, this trial used 3.16 Gy/fraction and our trial used 3 Gy/fraction in the intermediate-risk group and 3.33 Gy/fraction in the low-risk group.

The hypofractionated irradiation for prostate cancer (HYPRO) study and PROFIT study are two, large, randomized controlled trials comparing hypofractionated radiotherapy to conventional radiotherapy (Table 3). In the HYPRO study, the cumulative incidence of toxicity events was reported: acute grade ≥ 2 GU toxicity was 57.8% versus 60.5%, respectively and acute GI toxicity was 31.2% versus 42%. The cumulative incidence of grade ≥ 2 acute gastrointestinal toxicity was significantly higher in patients given hypofractionation than in those given standard fractionation [24]. In PROFIT, acute grade ≥ 2 GU toxicity was 31% for both standard and short fractionation regimens, and the acute GI toxicity was 10.5% versus 16.7%, respectively. At the end of the PROFIT study period, the incidence of grade ≥ 2 acute gastrointestinal toxicity was significantly higher in patients given hypofractionation than in those given standard fractionation [14]. Though it is difficult to draw firm conclusions based on comparisons across trials, the results of our study seem to suggest that pure hypofractionation may be equivalent or better than accelerated hypofractionation for providing favorable rates of acute toxicity.

Late toxicity

The concept of pure hypofractionation would suggest mathematically that late toxicity could be reduced, particularly late rectal toxicity. The results of the large multi-center PROFIT trial, as well as a recent single-center trial out of Princess Margaret Hospital (PMH) in Toronto, suggest that hypofractionation can lead to low rates of late toxicity (Table 3). IGRT was mandated in PROFIT, and dose volume histogram (DVH) constraints for organs-at-risk were tight, which may have contributed to the low toxicity rates achieved in these trials. Nevertheless, the PROFIT trial found an improved incidence of late GI toxicity in their short arm (i.e. hypofractionated) compared to conventional fractionation (8.9% vs. 13.9%, respectively). Both the short arm and standard arm had late GU toxicity ≥ grade 2 of 22% [14]. In the PMH trial, the incidence of late toxicity was low, demonstrating five-year late GI toxicity grade ≥ 2 of 4% and 21% and five-year late GU toxicity grade ≥ 2 of 9% and 4% for the 60 Gy and 66 Gy groups, respectively [26]. With our trial of pure hypofractionation, late toxicity rates were low (Table 2). Furthermore, 66% of the patients in our trial were treated with 3D-CRT and 34% with IMRT, whereas in the PROFIT and PMH studies, all patients were treated with IMRT. It is interesting that despite the use of 3D-CRT, pure hypofractionation still leads to relatively low rates of late toxicities. These findings might suggest that pure hypofractionated radiotherapy could be used to mitigate the issue of higher late toxicity rates that have been observed in other trials of accelerated hypofractionated radiotherapy [8,10,16,24,27-28]. Of course, factors other than overall treatment time may have contributed to our low rates of late toxicity.

In our trial, the five-year cumulative incidence of > grade 2 GU and GI toxicity (Figure 1) were 7.9% and 6.7%, respectively. The hypofractionated irradiation for prostate cancer (HYPRO) trial reported higher rates of grade ≥ 2 late toxicity in their hypofractionated arm compared to conventional fractionation. The three-year cumulative incidence of GI toxicity was 21.9% and 17.7% for hypofractionation versus conventional fractionation, respectively. The three-year incidence of GU toxicity was 41.3% and 39.0%, respectively [24]. The conventional or hypofractionated high dose intensity modulated radiotherapy for prostate cancer (CHHiP) trial (Table 3) compared groups treated with 74 Gy/37 fractions, 60 Gy/20 fractions, and 57 Gy/19 fractions, and the five-year cumulative incidence rates of late grade ≥2 GI toxicity were 13.7%, 11.9%, and 11.3%, respectively, and the rates of grade ≥ 2 GU toxicity were 9.1%, 11.7%, and 6.6%, respectively [10,29]. In the London study, late grade 2 and 3 toxicity for GU was 14% and 5%, and GI toxicity was 25% and 3%. Of note, acute GI toxicity ≥ grade 2 was shown to be a predictor of late toxicity grade ≥ 2 (p < 0.001) consistent with the theory of consequential late injury where acute normal tissue damage does not have sufficient time to repair [16,18-19].

Biochemical control

With a median follow-up of 6.5 years, this study reported five-year FFBF rates (Figure 2B) of 85% for low risk (50 Gy in 15 fractions over seven weeks) and 81% for Intermediate risk (60 Gy in 20 fractions over eight weeks). With a median follow-up of six years, PROFIT reported five-year biochemical disease-free survival rates of 85% for both the short fractionation arm and standard fractionation [14]. The CHHiP trial (median follow-up 50.5 months) reported five-year FFBF rates of 88.3%, 90.6%, and 85.9% in the 74 Gy, 60 Gy, and 57 Gy treatment groups, respectively [10]. The PMH cohort study reported that FFBF at five years was 81% in the 60 Gy cohort and 88% in the 66 Gy cohort (median follow-up was 128 months for 60 Gy and 108 months for 66 Gy) [26]. These studies confirm a high rate of biochemical control with hypofractionated radiotherapy, but longer-term outcomes are required for this risk category of prostate cancer [30]. See Table 3 for a summary of biochemical control statistics from each trial.

Limitations

Our trial spanned a period where image guidance and the use of IMRT was being introduced. Sixty-six percent of the patients on our trial were treated with 3D-CRT. Newer IMRT techniques may reduce differences in toxicity between pure and conventional hypofractionation.

It is not possible to make direct comparisons of our results with other trials, so we must be cautious in our interpretations. Nonetheless, the low rates of toxicity observed here are promising and might encourage a further exploration of pure hypofractionation. The London study explored standard hypofractionation over four to five weeks, and there were similarities between our trial and the London trial in terms of technology, margin recipes, dose constraints, and patient characteristics [16]. A comparison to this study might allow us to explore whether pure hypofractionation provides similar biochemical control but with lower toxicity. In particular, is pure hypofractionation possibly better for those at higher risk of a rectal consequential late injury? This difference is wide enough that a relatively small trial would have sufficient statistical power to detect a difference.

## Conclusions

Pure hypofractionation is feasible and may provide a simple method to minimize the risk of toxicity. The five-year biochemical control rates are similar, and the acute toxicity appears to be lower compared to published studies. Although not randomized, this trial suggests that pure hypofractionation could be considered a reasonable alternative in patients who are risk-averse, with higher risk dosimetry, with a higher risk for rectal toxicity, or elderly patients. Pure hypofractionation should be further tested in a randomized control trial.
